# Efficacy and safety of recombinant porcine factor VIII in Japanese patients with acquired hemophilia A

**DOI:** 10.1007/s12185-024-03823-y

**Published:** 2024-08-19

**Authors:** Yoshinobu Seki, Yoshiyuki Ogawa, Takahide Kikuchi, Emiko Sakaida, Yuki Mizuta, Tadayuki Kitagawa, Kazuhiko Takemura, Yasuo Miyaguchi, Keiji Nogami, Tadashi Matsushita

**Affiliations:** 1 Department of Hematology, Niigata University Medical and Dental Hospital, Niigata, Japan; 2 Department of Hematology, Niigata Cancer Center Hospital, Niigata, Japan; 3 Department of Hematology, Gunma University Hospital, Gunma, Japan; 4 Division of Hematology, Department of Internal Medicine, Saiseikai Central Hospital, Tokyo, Japan; 5 Department of Hematology, Chiba University Hospital, Chiba, Japan; 6 Takeda Pharmaceutical Company Limited, Osaka, Japan; 7 Takeda Pharmaceutical Company Limited, Tokyo, Japan; 8 Department of Pediatrics, Nara Medical University Hospital, Nara, Japan; 9 Department of Blood Transfusion, Nagoya University Hospital, Nagoya, Japan

**Keywords:** Acquired hemophilia A, Factor VIII deficiency, Japanese patients, Recombinant porcine factor VIII

## Abstract

Acquired hemophilia A (AHA) is a rare bleeding disorder caused by autoantibodies inhibiting human factor VIII (hFVIII). This phase II/III open-label study evaluated the safety and efficacy of recombinant porcine factor VIII (rpFVIII, susoctocog alfa) in adults with AHA and severe bleeding episodes in Japan (NCT04580407). The initial rpFVIII dose was 200 U/kg, with subsequent doses based on clinical measures including plasma FVIII activity. The primary efficacy endpoint was the proportion of severe bleeding episodes with a positive response to rpFVIII therapy 24 h after treatment initiation. Five patients were eligible for, and completed, rpFVIII treatment (age group: 60s–80s; median hFVIII inhibitor: 52 BU/mL; porcine FVIII [pFVIII] inhibitor: 3/5 patients). The median (range) total dose/patient was 548.4 (198–1803) U/kg with a median 3.0 infusions/patient. All patients responded positively to rpFVIII therapy at 24 h regardless of baseline pFVIII inhibitor status. rpFVIII treatment was well tolerated with no adverse events of special interest such as thromboembolic events or de novo pFVIII inhibitors. This study supports the use of rpFVIII as a novel therapy in the clinical management of patients with AHA in Japan. rpFVIII was approved for treating bleeding episodes in adults with AHA in Japan in 2024.

**Supplementary Information:**

The online version contains supplementary material available at 10.1007/s12185-024-03823-y.

## Introduction

Acquired hemophilia A (AHA) is a very rare bleeding disorder with an incidence of approximately 1.3–1.5 individuals per million per year [1–5]. It is caused by the development of autoantibodies to human factor VIII (hFVIII) and manifests as spontaneous, often severe or life-threatening bleeding at anatomically diverse sites [3]. It is typically seen in people aged between 70 and 80 years [3]. In 2022, there were 296 reported cases of AHA in Japan [6]. Two regional studies have reported mortality of 13.8% (8/58 patients over 17 months) [7] and 25% (10/40 patients over 3 years) [8] in patients with AHA in Japan, in line with global estimates [9].

Current treatment recommendations for AHA involve the use of hemostatic therapies to control bleeding, alongside immunosuppressive therapies to suppress the production of anti-FVIII autoantibodies (also known as inhibitors) [10, 11]. For patients with congenital hemophilia A without inhibitors, the most effective approach for the treatment of acute bleeding episodes is hFVIII replacement therapy [12, 13]. The presence of autoantibodies to hFVIII in patients with AHA inhibits the activity of hFVIII agents, which means this is not a suitable treatment option. In Japan, the standard of care for achieving hemostasis in patients with AHA is to use bypassing agents such as recombinant activated factor VII (rFVIIa), activated prothrombin complex concentrate (aPCC), and plasma-derived activated factor VII/factor X complex concentrate (pd-FVIIa/FX), the latter of which is only available in Japan [10]. However, the use of bypassing agents is associated with a risk of thrombosis, which increases both as patients get older and in the presence of certain comorbidities (such as collagen vascular disorders and malignancy) [14]. In addition, there is no standard laboratory assay to monitor treatment with bypassing agents [15, 16].

In the 1980s, plasma-derived porcine factor VIII (pFVIII; Hyate:C^®^) was used successfully to achieve hemostasis in patients with inhibitory antibodies to hFVIII, because anti-hFVIII antibodies generally have low immunological cross-reactivity with pFVIII [17]. However, pFVIII was withdrawn from use in 2004 owing to viral safety concerns [18–21]. Recombinant pFVIII (rpFVIII) is a purified B-domain deleted form of pFVIII expressed as a glycoprotein using a well-defined genetically engineered baby hamster kidney (BHK) cell line. The structure of rpFVIII is sufficiently similar to hFVIII that it can temporarily replace the inhibited endogenous FVIII that is needed for effective hemostasis, yet is different enough to be less susceptible to inactivation by circulating inhibitory antibodies [22]. rpFVIII (OBIZUR^®^) was approved for use by the US Food and Drug Administration in 2014 [23] and by the European Medicines Agency in 2015 [24] for the on-demand treatment and control of bleeding episodes in adults with AHA. This approval was based on data from a phase II/III open-label multicenter study carried out in the USA, UK, India, and Canada, in which 24 out of 28 patients with AHA achieved control of the qualifying bleed following treatment with rpFVIII (NCT01178294) [25]. The study authors concluded that rpFVIII had a good safety profile and was effective in treating bleeding episodes in people with AHA, even in patients with baseline anti-pFVIII antibodies. The authors also highlighted the clinical advantage of being able to adjust the dose and frequency of rpFVIII based on factor VIII activity (FVIII:C) [25].

The aim of our study was to evaluate the safety and efficacy of rpFVIII in Japanese patients with AHA.

## Methods

### Study design and patient population

This was a phase II/III, multicenter, prospective, open-label, non-controlled study to evaluate the efficacy and safety of rpFVIII for the treatment of severe bleeding episodes in Japanese patients with AHA (ClinicalTrials.gov ID: NCT04580407). The study protocol, informed consent form, and all amendments were reviewed and approved by the local institutional review boards of each investigator site before study initiation. The study was conducted in accordance with the Declaration of Helsinki and the principles and guidelines described in the study protocol. Informed consent was received from all patients in this study.

Eligible patients included men and women aged at least 18 years who were suspected of having, or had previously received, an AHA diagnosis (based on clinical evaluation and laboratory testing) and who presented with a severe bleeding episode (e.g., threatening vital organ function, requiring a blood transfusion, compromising muscle viability or neurovascular integrity, or affecting a major joint). Once a diagnosis of AHA had been confirmed, patients were able to start treatment with rpFVIII. Prior treatment with rFVIIa, aPCC, and pd-FVIIa/FX was not an exclusion criterion, provided that a washout period was allowed (3 h, 6 h, or 8 h, respectively) before the initial rpFVIII infusion.

### Study treatments and administration

rpFVIII was administered at an initial dose of 200 U/kg. The need for additional doses was determined by the investigator based on clinical bleeding status (reviewed every 6–12 h as per expert consensus recommendations) [26] and clinical laboratory evaluation (FVIII:C [measured with a one-stage clotting assay using the World Health Organization hFVIII plasma standard], activated partial thromboplastin clotting time [aPTT], hemoglobin [Hgb] and hematocrit [Hct] at 30 min, 8 h, 16 h, and 24 h after the first infusion, then every 12 h until 72 h, and then every 24 h until the end of infusion/study). If additional doses of rpFVIII were administered, FVIII:C, aPTT, Hgb, and Hct were also measured at each subsequent dose until 24 h after the dose was administered. Additional doses of rpFVIII were administered as frequently as every 4–12 h with dose and frequency determined based on the post-infusion FVIII:C result and the target FVIII:C. For bleeds of particular clinical concern (e.g., severe mucosal, intracranial, retro- or intra-abdominal, genitourinary, neck, traumatic, or postoperative bleeds), the target trough FVIII:C was at least 80% for the first 24 h. For all other severe bleeding episodes (e.g., joint, muscle, soft tissue) in the first 24 h and all bleeding episodes after the first 24 h, the target trough FVIII:C was at least 50%. The dose of rpFVIII could not exceed 800 U/kg every 4 h. Treatment with rpFVIII was continued until bleeding was successfully controlled, until the investigator concluded a lack of efficacy, or until the patient withdrew from the study.

### Efficacy endpoints

The primary efficacy endpoint was the proportion of severe bleeding episodes with a demonstrated positive response to rpFVIII therapy at 24 h after the initiation of treatment using a well-defined four-point ordinal scale (Supplementary Table S1). A positive response was defined as effective or partially effective assessment of efficacy. Control of bleeding was evaluated based on obvious blood loss (external blood loss and bodily fluids), hematology results, blood transfusion and blood component requirements, physical or technological examination of the bleeding site, neurological examination, and imaging studies. Patients who experienced a therapeutic response to rpFVIII were eligible for treatment with rpFVIII for any subsequent major bleeding episode but outcomes for these bleeding episodes were not part of the primary efficacy analysis. Re-bleeding was considered to have occurred if there was bleeding from a previously successfully controlled site within 2 weeks of the last dose of rpFVIII. In this case, the event was recorded as an adverse event (AE) and treated appropriately. Key secondary efficacy endpoints included: the overall proportion of severe bleeding episodes successfully controlled with rpFVIII therapy; the proportion of bleeding episodes responsive to rpFVIII therapy at 30 min, 8 h, and 16 h after the initiation of therapy, and every 14 days until the end of the study (90 days after the final dose of rpFVIII for initial bleeding, to cover the average time to remission of AHA [3, 27] including resolution of other bleeding besides initial bleeding); and the frequency, total dose, and total number of infusions of rpFVIII required to successfully control qualified bleeding episodes.

### Safety endpoints

Safety endpoints included AEs, serious adverse events (SAEs), clinical laboratory measurements, vital signs, and the presence of anti-BHK cell antibodies. AEs of special interest included hypersensitivity reactions, the development of de novo inhibitors to pFVIII, anamnestic reactions with an increase of inhibitor titer to pFVIII and/or hFVIII, and thromboembolic events.

### Statistical analyses

The planned total sample size for this study was five patients, which was based on feasibility considerations given the low incidence of AHA in Japan. The primary efficacy endpoint was calculated as the proportion of patients with a positive response to rpFVIII therapy at 24 h post-treatment and the corresponding exact two-sided Clopper–Pearson 95% confidence intervals (CIs). Patients who had hemostatic response and stopped treatment because bleeding was controlled were assumed to be responders at the 24-h assessment time point.

## Results

### Patient demographics and baseline characteristics

In total, six patients signed the informed consent form, and five patients were eligible to receive treatment. Of the five patients who entered the study treatment period, all patients completed rpFVIII treatment for the initial qualified bleeding episode. One patient (patient 3) discontinued the study owing to the need to receive prophylactic treatment with emicizumab. Three men and two women were included in the study. All patients were aged in their 60s to 80s, and the median (range) weight was 51.7 (46.7–65.7) kg (Table 1). Four patients received a diagnosis of AHA at this admission and one patient received a diagnosis 33 months previously. The median (range) hFVIII inhibitor concentration at baseline was 52.0 (7–150) Bethesda units (BU)/mL. Three out of five (60%) patients had detectable pFVIII inhibitors at baseline and the median (range) pFVIII inhibitor titer for all five patients was 0.9 (0.0–9.3) BU/mL. Three patients had a history of rFVIIa treatment, and one patient had a history of pd-FVIIa/FX treatment in the 6-month period before receiving rpFVIII. All patients used immunosuppressive medicine concomitantly (prednisolone was used by three patients for AHA, by one patient for multiple uses, and by one patient for systemic lupus erythematous as a comorbidity; Supplementary Table S2). No patient received a blood transfusion in this study or in the 6 months before the study started.

**Table 1:** Patient demographics and baseline characteristics

Characteristic	Patient					Mean (SD)	Median (range)
	1	2	3	4	5		
Sex	Female	Female	Male	Male	Male	–	–
Age group (decade)^a^	80s	60s	80s	70s	70s	–	–
Weight (kg)	48.3	46.7	51.7	65.7	54.7	53.4 (7.5)	51.7 (46.7–65.7)
Time since first diagnosis of AHA (months)	0	33	0	0	0	6.6 (14.6)	0 (0–33)
Concomitant medication	Prednisolone	Prednisolone	Prednisolone	Prednisolone	Prednisolone	–	–
History of bypassing agents in the 6 months before rpFVIII	pd-FVIIa/FX	rFVIIa	N/A	rFVIIa	rFVIIa	–	–
FVIII:C at baseline^b^ (%)	6.4	< 1.0	< 1.0	< 1.0	0.9	1.5 (2.8)	0.0 (0.0–6.4)
hFVIII inhibitor concentration at baseline (BU/mL)	25	150	52	7	54	57.6 (55.2)	52.0 (7–150)
pFVIII inhibitor concentration at baseline^b^ (BU/mL)	8.7	9.3	0.9	< 0.6	< 0.6	3.8 (4.8)	0.9 (0.0–9.3)
Hemoglobin at baseline (g/L)	77	79	106	110	81	90.6 (16.0)	81 (77–110)
Bleeding episode history							
Site	Skin, muscle	Skin, muscle, soft tissue	Skin, venipuncture site, mucosal	Skin, muscle	Skin, soft tissue, hematuria, CNS	–	–
Cause	Spontaneous	Injury	Spontaneous	Spontaneous	Spontaneous	–	–
Severity	Severe	Severe	Severe	Severe	Severe	–	–

Baseline values shown in this table were obtained at the initial dose of rpFVIII (or at screening if initial dose data were not available). No patient received a blood transfusion in this study or in the 6 months before the study started

*AHA* acquired hemophilia A, *aPTT* activated partial thromboplastin time, *BU* Bethesda unit, *CNS* central nervous system, *FVIII:C* FVIII activity, *hFVIII* human factor VIII, *N/A* not applicable, *pd-FVIIa/FX* plasma-derived activated factor VII/factor X complex concentrate, *pFVIII* porcine factor VIII, *rFVIIa* recombinant activated factor VII, *rpFVIII* recombinant porcine factor VIII, *SD* standard deviation

^a^Actual values not shown to prevent patient identification

^b^For the calculation of descriptive statistics, values stated as < 1.0 (FVIII:C) or < 0.6 (pFVIII inhibitor) were considered to be 0.0

### Efficacy

All five patients (100%, 95% CI 47.8–100.0) were assessed by the investigators to have achieved the primary efficacy endpoint (severe bleeding episodes with a positive response to rpFVIII therapy at 24 h after the initiation of treatment; Table 2).

**Table 2:** Response to rpFVIII infusion at key assessment time points post-treatment

Assessment time point	Response to rpFVIII therapy after the initiation of treatment		Total number of patients	Exact two-sided Clopper–Pearson 95% CI
	Positive, *n* (%)	Negative, *n* (%)		
30 min	4 (80)	1 (20)	5	28.4, 99.5
8 h	5 (100)	0 (0)	5	47.8, 100.0
16 h	4 (100)	0 (0)	4	39.8, 100.0
**24 h**	**5 (100)**	**0 (0)**	**5**	**47.8, 100.0**
Follow-up at 14 days	5 (100)	0 (0)	5	47.8, 100.0
Follow-up at 28 days	4 (100)	0 (0)	4	39.8, 100.0
Follow-up at 42 days	4 (100)	0 (0)	4	39.8, 100.0
Follow-up at 56 days	4 (100)	0 (0)	4	39.8, 100.0
Follow-up at 70 days	4 (100)	0 (0)	4	39.8, 100.0
End of study period	4 (100)	0 (0)	4	39.8, 100.0

Data show responses to rpFVIII infusion and the corresponding exact two-sided Clopper–Pearson 95% CIs. The primary efficacy endpoint was the proportion of patients with a positive response to rpFVIII therapy at 24 h (shown in bold). Note: eligible patients who withdrew from the treatment at an earlier time point were assumed to be non-responders at the 24-h assessment time point. Patients who had a hemostatic response and stopped treatment because bleeding was controlled were assumed to be responders at the 24-h assessment time point. One patient who was evaluated for the 16-h time point had an assessment time that was outside the allowance window for evaluation stated in the statistical analysis plan. One patient was not evaluated after the 14-day time point due to discontinuing the study before the 28-day time point

*CI* confidence interval, *rpFVIII* recombinant porcine factor VIII

The proportion of patients who had a response to rpFVIII therapy at designated assessment time points after the initiation of therapy is summarized in Table 2. Four out of five patients showed a positive response at 30 min after the initial dose of rpFVIII. All patients whose responses were assessed showed a positive response to rpFVIII at 8 h, 16 h, and 24 h after the initial dose, at all assessed follow-up visits, and at the end of study visit. No patient experienced a re-bleed in this study. Overall, all five patients were assessed by the investigator to have successfully controlled their severe bleeding episode with rpFVIII treatment in this study.

Total dose, frequency of infusions, and total number of infusions of rpFVIII required to control severe bleeding episodes successfully are summarized in Table 3. The median (range) total dose of rpFVIII per patient was 548.4 (198–1803) U/kg. Individual patient responses to treatment and dosage information are shown in Table 4. One patient received one infusion and four patients received three infusions of rpFVIII. The median number of total infusions per patient was 3.0 infusions and the median (range) number of infusions was 1.5 (1–3) infusions/day. Compliance for rpFVIII dosing was 100% in all five patients.

**Table 3:** Total dose, frequency, and number of infusions of rpFVIII required to successfully control qualified bleeding episodes in the initial treatment period

	Number of patients	Mean (SD)	Median (range)
Total dose per patient (U/kg)	5	770.5 (631.35)	548.4 (198–1803)
Total number of infusions per patient	5	2.6 (0.89)	3.0 (1–3)
Average number of infusions per day	5	1.8 (1.12)	1.5 (1–3)

*rpFVIII* recombinant porcine factor VIII, *SD* standard deviation

**Table 4:** Individual patient responses and treatment dose/timings

Patient	Efficacy assessment^a^ at 24 h after the first dose	Peak FVIII:C within 24 h of treatment (%)	Dose of rpFVIII (U/kg)^b^	Treatment start time^b^
1	Effective	80.7	197	0 h
			104	+ 6 h 0 min
			104	+ 12 h 2 min
2	Effective	65.7	200	0 h
			800	+ 5 h 49 min
			803	+ 11 h 19 min
3	Effective	187.2	203	0 h
			300	+ 8 h 49 min
			397	+ 24 h 34 min
4	Effective	369.0	198	0 h
5	Effective	241.2	201	0 h

*FVIII:C* FVIII activity, *h* hour, *min* minute, *rpFVIII* recombinant porcine factor VIII, *U* units

^a^Investigator assessment of hemostatic response to rpFVIII using a four-point ordinal scale (effective, partially effective, poorly effective, not effective)

^b^Only doses administered in the first 24 h after first dose are listed

**Fig. 1:**
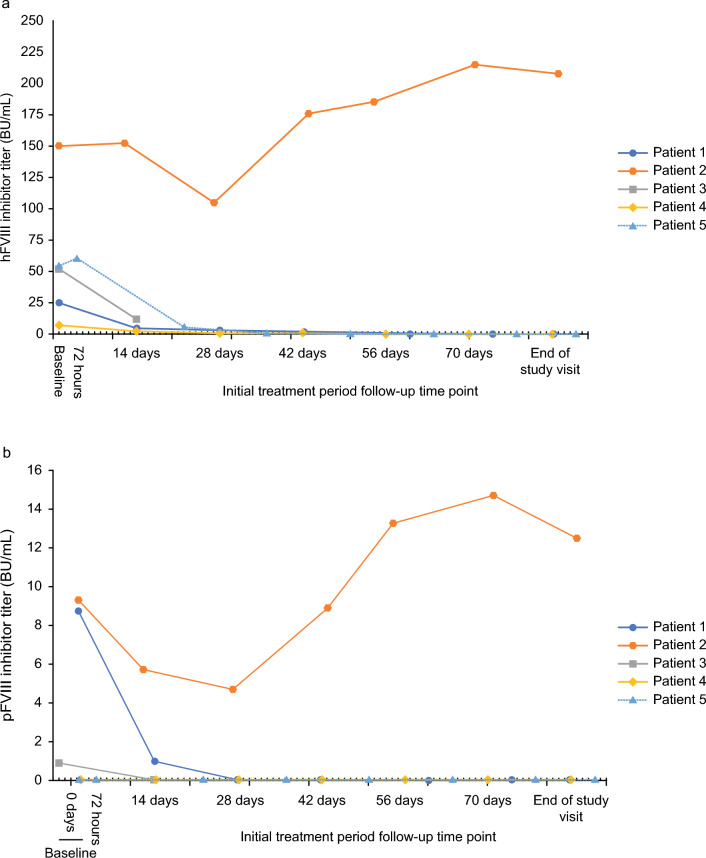
Individual patient inhibitor titers against hFVIII and pFVIII over the entire treatment period. Data show individual patient inhibitor titers against **a** hFVIII and **b** pFVIII. For pFVIII inhibitor titers, the lower limit of quantification was > 0.6 BU/mL (shown as 0.0 BU/mL in this figure). Baseline values were obtained on study day 1 (either at screening or at initial treatment dose) for all patients except for patient 3, whose baseline pFVIII measurement was obtained on study day − 2 (at screening). *BU* Bethesda unit, *hFVIII* human factor VIII, *pFVIII* porcine factor VIII

Individual patient inhibitor titers against hFVIII and pFVIII throughout the study are shown in Fig. 1. Although three patients had detectable pFVIII inhibitors before the initial dose, all five patients were assessed to have a positive response to rpFVIII at 24 h after initiation of treatment and achieved eventual control of the bleeding episode. No patient developed de novo pFVIII inhibitors or anamnestic reactions with an increase of inhibitor titers against pFVIII and/or hFVIII. One patient (patient 2) did develop an increase of inhibitor titers against pFVIII and hFVIII from baseline, but as this occurred 42 days after the end of the rpFVIII infusion, it was not considered an anamnestic reaction to rpFVIII. This patient was taking prednisolone (Supplementary Table S2) at a lower dose (15 mg) than that recommended by the Japanese AHA guidelines for their weight (46.7 kg) [10], which may have reduced the efficacy of the prednisolone and resulted in an increase in hFVIII inhibitor titers. These inhibitors may have cross-reacted with pFVIII and caused a similar increase in the pFVIII inhibitor titer. The two patients in whom pFVIII inhibitors were not detected at baseline did not develop pFVIII inhibitors after initiation of treatment.

**Fig. 2:**
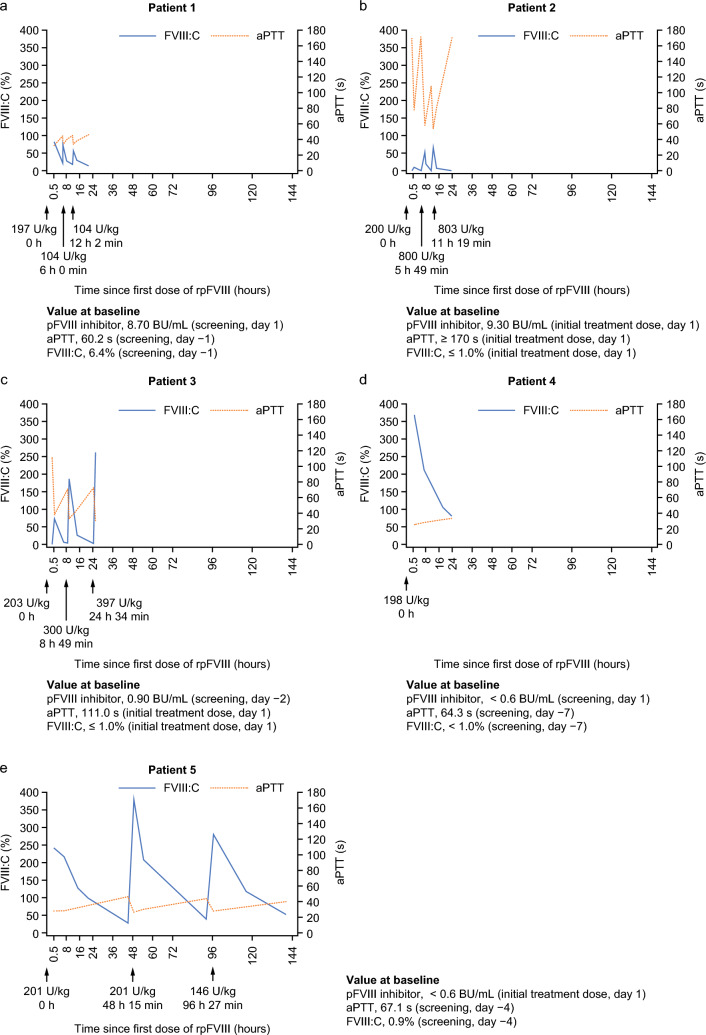
Individual patient FVIII:C and aPTT over the entire treatment period. Dose (U/kg) and timing of rpFVIII treatment relative to the first dose of rpFVIII (0 h) are shown by arrows. For FVIII:C, the lower limit of quantification was ≤ 1% (shown as 0% in this figure). For aPTT, the upper limit of quantification was ≥ 170 s (shown as 170 s in this figure). Values at baseline (either at screening or initial treatment dose) are also shown for pFVIII inhibitor concentrations, aPTT and FVIII:C. *aPTT* activated partial thromboplastin time, *BU* Bethesda unit, *d* day, *FVIII:C* factor VIII activity, *h* hour, *min* minute, *pFVIII* porcine factor VIII, *rpFVIII* recombinant porcine factor VIII, *s* second

Individual patient plots of FVIII:C and aPTT with rpFVIII dosing information over the study are shown in Fig. 2 and summarized in Table 4. Overall, FVIII:C increased following each rpFVIII infusion, although the increase was variable between patients. For example, one patient received a single infusion of rpFVIII 198 U/kg, and immediately had FVIII:C exceeding 300%. The FVIII:C in this patient remained over 50% during the 24 h period that followed (Fig. 2d). In contrast, another patient had FVIII:C of approximately 10% immediately after their first dose, which then fell to 1% by 6 h after the first infusion. This patient had pFVIII inhibitors at baseline, and subsequently received two further infusions at the maximum possible dose (800 U/kg and 803 U/kg) (Fig. 2b). Overall, the median (range) duration of rpFVIII treatment was 1.0 (1–5) days. The patient who received the single infusion had a 1-day treatment duration, while of the four patients who received three infusions, three patients received second and third infusions within approximately 24 h following the initial dose of rpFVIII (Fig. 2a–c), and one patient received second and third infusions on day 3 and day 5, respectively (Fig. 2e). After each infusion, aPTT tended to shorten as FVIII:C increased. In patients with pFVIII inhibitors at baseline (Fig. 2a–c), the increase in FVIII:C after the first infusion of rpFVIII was smaller than the increase seen in patients without pFVIII inhibitors (Fig. 2d and e). For four out of five patients, the maximum FVIII:C recorded in the 24 h following the first infusion was at least 50%, even for patients with pFVIII inhibitors at baseline.

### Safety

In total, 49 AEs were reported in five patients, most of which were mild to moderate in severity. Two severe AEs (urinary tract infection and subcutaneous hemorrhage) were reported in one patient, with neither AE considered related to rpFVIII treatment. AEs by system organ class reported in at least three patients were skin and subcutaneous tissue disorders (reported in four patients) and gastrointestinal disorders (reported in three patients).

Five SAEs were reported in two patients in this study. Patient 2 experienced three SAEs (subcutaneous hemorrhage, hemorrhagic diathesis, and urinary tract infection), which were considered by the investigator to be unrelated to rpFVIII treatment. Patient 3 experienced two SAEs (cryoglobulinemia and central hypothyroidism), which were both considered by the investigator to be related to rpFVIII treatment because a causal relationship could not be completely denied. The sponsor considered it unlikely that both events were related to rpFVIII because the patient had a medical history of autoimmune disease (seronegative arthritis) (Supplementary Table S3) and there was no compelling evidence that directly linked the study medication to the events. This patient had to discontinue the study because treatment with emicizumab was required. Their condition deteriorated, and the patient died of general debility 3 months later. No other AEs (serious or nonserious) were considered by the investigator to be related to rpFVIII treatment. There were no reports from the investigators of hypersensitivity reactions or thromboembolic events, and no patient developed anti-BHK antibodies. There were also no clinically significant changes in laboratory parameters or vital signs throughout the study. The sponsor classified one AE of erythema as an AE of special interest (hypersensitivity reactions), but this was not considered related to the study drug. No deaths or AEs leading to study drug discontinuation were reported during the AE collection period in this study.

## Discussion

This phase II/III study was the first clinical trial to assess the safety and efficacy of rpFVIII treatment in Japanese patients with AHA. All patients in this study, including those with pFVIII inhibitors at baseline, responded positively to rpFVIII therapy (defined as an effective or partially effective assessment of efficacy) within 24 h after treatment initiation. Treatment with rpFVIII was well tolerated and no patients had thromboembolic events. Results from our study were comparable to those obtained in the global phase II/III study of rpFVIII [25].

In the three patients with pFVIII inhibitors at baseline in our study, the increase in FVIII:C following the first dose of rpFVIII was smaller than in patients without pFVIII inhibitors. However, all three patients did achieve FVIII:C above 50% within the first 24 h, requiring either one or two doses of rpFVIII to do so. After achieving FVIII:C above 50%, activity did fall back down below 50% in each patient and repeat administration of rpFVIII was required. This result demonstrates that repeated rpFVIII administration can sufficiently neutralize any pFVIII inhibitors present and enable the remaining pFVIII to function effectively. It is important to note that none of the patients in our study developed de novo pFVIII inhibitors.

The aPTT during treatment with rpFVIII tended to be inversely correlated with FVIII:C. Generally, aPTT reached a plateau within the normal range when FVIII:C was 40% or higher, suggesting that aPTT could not be used alone as a substitute for FVIII:C. This is consistent with results from an ex vivo, retrospective study on the complexities of using the aPTT assay to monitor FVIII deficiencies (among other factors) in patients with hemophilia [28]. Further investigation is needed to understand the relationship between aPTT and FVIII:C during rpFVIII treatment.

In our study, dose and dosing frequency varied widely. One patient received a single infusion of rpFVIII and immediately recorded FVIII:C above 300%, which remained over 50% during the 24-h period that followed. In contrast, another patient, who had pFVIII inhibitors at baseline, had FVIII:C of about 10% immediately after their first dose, which decreased to 1% by 6 h after the first infusion. This patient then received two further infusions at the maximum possible dose. This variation among patients demonstrates that personalized therapy, in which the dose and frequency of rpFVIII treatment is adjusted based on individual FVIII activity and clinical symptoms, can contribute to successful hemostasis.

Four out of five patients had received bypassing agents in the 6 months before rpFVIII administration; a positive response to rpFVIII was confirmed in all patients regardless of their previous use of bypassing agents. Although patient numbers were small in our study, this result suggests that rpFVIII can exert a sufficient hemostatic effect as both first-line therapy and second-line therapy when the efficacy of bypassing agents is insufficient. This conclusion is supported by results from the global phase II/III study on rpFVIII, which showed that rpFVIII treatment was successful in 24/28 (85.7%) patients regardless of previous treatment with another hemostatic agent [25].

In our study, rpFVIII was well tolerated with a similar safety profile to that reported previously [25]. The only AE of special interest was a case of erythema, which was classified as a hypersensitivity reaction by the sponsor but not the investigators. There were no other AEs of special interest, including development of de novo inhibitors to pFVIII, anamnestic reactions, or thromboembolic events.

Limitations of this study include the small sample size. This is a consequence of the very low incidence of AHA in Japan and thus the limited number of patients who could be recruited. The study did not include a treatment comparator arm for the same reason. Comparative data from larger studies would be beneficial to support future treatment decisions for patients with AHA in Japan.

In conclusion, this is the first published study to investigate the efficacy and safety of rpFVIII in Japanese patients with AHA. rpFVIII treatment was well tolerated and effectively controlled severe bleeding events in all five patients with AHA, which is consistent with data from the global study. rpFVIII was approved for the treatment of bleeding episodes in adults with AHA in Japan in 2024. The use of rpFVIII therapy with concurrent FVIII:C monitoring could contribute to rapid and reliable hemostasis for serious bleeding episodes in patients with AHA in Japan.

## Supplementary Information

Below is the link to the electronic supplementary material.

Supplementary file1 (DOCX 28 KB)

## Data Availability

Takeda does not plan to share data supporting the results reported in this article because there is a reasonable likelihood that individual patients could be re-identified (due to the limited number of study participants).
