# Supplementary material for: Efficacy and safety of recombinant porcine factor VIII in Japanese patients with acquired hemophilia A
Source: Int J Hematol. 2024 Aug 19;120(4):482–91. doi: 10.1007/s12185-024-03823-y (PMC11415465; doi:10.1007/s12185-024-03823-y)
Supplement: Supplementary file 1 — Supplementary file1 (DOCX 28 KB) [file 12185_2024_3823_MOESM1_ESM.docx]

**Supplementary Information**

## Table S1 Investigator assessment of hemostatic response to rpFVIII: four-point ordinal scale

| Assessment of efficacy | Control of bleeding | Clinical assessment | FVIII:C | Response |
| --- | --- | --- | --- | --- |
| Effective | Bleeding stopped | Clinical control | ≥ 50% | Positive |
| Partially effective | Bleeding reduced | Clinical stabilization or improvement or alternative reason for bleeding | ≥ 20% | Positive |
| Poorly effective | Bleeding slightly reduced or unchanged | Not clinically stable | < 50% | Negative |
| Not effective | Bleeding worsening | Clinically deteriorating | < 20% | Negative |
| If there was a discrepancy between the control of bleeding/clinical assessment and the FVIII:C levels, the control of bleeding/clinical assessment was used to determine the assessment of efficacy.  *FVIII:C* FVIII activity, *rpFVIII* recombinant porcine factor VIII | | | | |

## Table S2 Concomitant medications

|  | |  |  | |  | |  | | **Study day** | |
| --- | --- | --- | --- | --- | --- | --- | --- | --- | --- | --- |
| **Patient** | **Preferred**  **medication name** | | | **Dose (mg)** | | **Frequency** | | **Route** | **Start** | **End** |
| 1 | Prednisolone | | | 25 | | QD | | Oral | 1 | 8 |
|  | Prednisolone | | | 10 | | BID | | Oral | 9 | 14 |
|  | Prednisolone | | | 17.5 | | QD | | Oral | 15 | 21 |
|  | Prednisolone | | | 15 | | QD | | Oral | 22 | 28 |
|  | Prednisolone | | | 12.5 | | QD | | Oral | 29 | 60 |
|  | Prednisolone | | | 5 | | BID | | Oral | 61 | 84 |
| 2 | Prednisolone | | | 15 | | QD | | Oral | Before initial dosing | Ongoing |
| 3 | Prednisolone | | | 50 | | QD | | Oral | Before initial dosing | Ongoing |
| 4 | Prednisolone | | | 50 | | QD | | Intravenous | Before initial dosing | 4 |
|  | Prednisolone | | | 50 | | QD | | Oral | 5 | 8 |
|  | Prednisolone | | | 40 | | QD | | Oral | 9 | 14 |
|  | Prednisolone | | | 30 | | QD | | Oral | 15 | 28 |
|  | Prednisolone | | | 25 | | QD | | Oral | 29 | 42 |
|  | Prednisolone | | | 20 | | QD | | Oral | 43 | 56 |
|  | Prednisolone | | | 15 | | QD | | Oral | 57 | 70 |
|  | Prednisolone | | | 12.5 | | QD | | Oral | 71 | Ongoing |
| 5 | Prednisolone | | | 100 | | QD | | Intravenous | Before initial dosing | 45 |
|  | Prednisolone | | | 90 | | QD | | Intravenous | 46 | 52 |
|  | Prednisolone | | | 80 | | QD | | Intravenous | 53 | 59 |
|  | Prednisolone | | | 70 | | QD | | Intravenous | 60 | 66 |
|  | Prednisolone | | | 30 | | BID | | Oral | 67 | 73 |
|  | Prednisolone | | | 25 | | BID | | Oral | 74 | 80 |
|  | Prednisolone | | | 20 | | BID | | Oral | 81 | 87 |
|  | Prednisolone | | | 15 | | BID | | Oral | 88 | Ongoing |

*BID* two times per day, *QD* daily

**Table S3** Concurrent medical conditions

| **Patient** | **Concurrent Medical Condition (Preferred Term)** |
| --- | --- |
| 1 | Angina pectoris, asthma, cardiac failure chronic, cholelithiasis, chronic kidney disease, constipation, depression, glossitis, hyperuricemia, insomnia, osteoporosis, spinal osteoarthritis |
| 2 | Anemia, hematoma, myasthenia gravis, osteoporosis, skin ulcer, systemic lupus erythematosus, urinary tract infection |
| 3 | Anemia, hepatic function abnormal, hypertension, hyperuricemia, lumbar spinal stenosis, renal impairment, seronegative arthritis |
| 4 | Benign prostatic hyperplasia, cardiac failure chronic, diabetes mellitus, hyperlipidemia, hypertension, hyperuricemia, psoriasis |
| 5 | Alcoholic liver disease, delirium, dementia, epilepsy, hepatic steatosis, hypokalemia, ischemic hepatitis, prerenal failure, rhabdomyolysis, skin erosion, tinea pedis, urinary tract infection |
